# Supplementary material for: A study protocol to characterise pathophysiological and molecular markers of rheumatic heart disease and degenerative aortic stenosis using multiparametric cardiovascular imaging and multiomics techniques
Source: PLoS One. 2024 May 13;19(5):e0303496. doi: 10.1371/journal.pone.0303496 (PMC11090351; doi:10.1371/journal.pone.0303496)
Supplement: Attachment — Submitted filename: Response to Reviewers.docx [file pone.0303496.s001.docx]

Mohanad Alkhodarithe,

Academic Editor,

PLOS ONE Journal.

28^th^ March 2024.

Dear Mohanad,

**RE: Resubmission of a manuscript reference PONE-D-24-0093**

Please find attached a revised study protocol manuscript for publication at PLOS ONE, originally titled “**A study protocol to characterise pathophysiological and molecular markers of rheumatic heart disease and degenerative aortic stenosis using multiparametric cardiovascular imaging and multi-omics techniques.**”

The editor’s and reviewers’ comments and suggestions were very insightful and have helped improve the quality of the manuscript. Revisions in the revised manuscript have been highlighted using track changes. We have also included the clean version of the revised manuscript which has also undergone substantial English language editing. Below is our item-by-item response to the editor’s and the reviewers’ comments and suggestions.

**Comments from Senior Staff Editor:**

We note that one or more reviewers has recommended that you cite specific previously published works. As always, we recommend that you please review and evaluate the requested works to determine whether they are relevant and should be cited. It is not a requirement to cite these works. We appreciate your attention to this request.

**Response: We have read the suggested articles and thank the reviewers for their suggestions. In the revised manuscript we have added some citations far and beyond.**

Journal requirements:

When submitting your revision, we need you to address these additional requirements.

1. Please ensure that your manuscript meets PLOS ONE's style requirements, including those for file naming. The PLOS ONE style templates can be found at

[https://journals.plos.org/plosone/s/file?id=wjVg/PLOSOne_formatting_sample_main_body.pdf](https://eur01.safelinks.protection.outlook.com/?url=https%3A%2F%2Furl.za.m.mimecastprotect.com%2Fs%2FuqknCRgKx1fvBJVk1fOwlyj&data=05%7C02%7Cmttdan008%40myuct.ac.za%7C323ec11e6bfa4539acb308dc49045d61%7C92454335564e4ccfb0b024445b8c03f7%7C0%7C0%7C638465530874751149%7CUnknown%7CTWFpbGZsb3d8eyJWIjoiMC4wLjAwMDAiLCJQIjoiV2luMzIiLCJBTiI6Ik1haWwiLCJXVCI6Mn0%3D%7C0%7C%7C%7C&sdata=TrGqcEcmf7j9w%2FprHorZkdAQE8FIDtHgzvaAhAY%2FeS8%3D&reserved=0) and <https://journals.plos.org/plosone/s/file?id=ba62/PLOSOne_formatting_sample_title_authors_affiliations.pdf>

**Response: We have edited the format of the manuscript's main body and the title and authors affiliations.**

2. Your ethics statement should only appear in the Methods section of your manuscript. If your ethics statement is written in any section besides the Methods, please delete it from any other section.

**Response: The extra ethics statement has been removed in the revised manuscript. It now only appears in the methods section.**

3. We note that Figures 1, 2 and 3 in your submission contain copyrighted images. All PLOS content is published under the Creative Commons Attribution License (CC BY 4.0), which means that the manuscript, images, and Supporting Information files will be freely available online, and any third party is permitted to access, download, copy, distribute, and use these materials in any way, even commercially, with proper attribution. For more information, see our copyright guidelines: [http://journals.plos.org/plosone/s/licenses-and-copyright](https://eur01.safelinks.protection.outlook.com/?url=https%3A%2F%2Furl.za.m.mimecastprotect.com%2Fs%2F2LWaCWnKDqi5G2kgKUpkZsM&data=05%7C02%7Cmttdan008%40myuct.ac.za%7C323ec11e6bfa4539acb308dc49045d61%7C92454335564e4ccfb0b024445b8c03f7%7C0%7C0%7C638465530874761899%7CUnknown%7CTWFpbGZsb3d8eyJWIjoiMC4wLjAwMDAiLCJQIjoiV2luMzIiLCJBTiI6Ik1haWwiLCJXVCI6Mn0%3D%7C0%7C%7C%7C&sdata=BnBNxDARJjAIMk0m85GIEOIlAHHWrxkO4eLjD3%2BCQAk%3D&reserved=0).

We require you to either (1) present written permission from the copyright holder to publish these figures specifically under the CC BY 4.0 license, or (2) remove the figures from your submission:

1. You may seek permission from the original copyright holder of Figures 1, 2 and 3 to publish the content specifically under the CC BY 4.0 license.

We recommend that you contact the original copyright holder with the Content Permission Form ([http://journals.plos.org/plosone/s/file?id=7c09/content-permission-form.pdf](https://eur01.safelinks.protection.outlook.com/?url=https%3A%2F%2Furl.za.m.mimecastprotect.com%2Fs%2FoQ-uCX6XE5tX01JyRI7qsio&data=05%7C02%7Cmttdan008%40myuct.ac.za%7C323ec11e6bfa4539acb308dc49045d61%7C92454335564e4ccfb0b024445b8c03f7%7C0%7C0%7C638465530874767327%7CUnknown%7CTWFpbGZsb3d8eyJWIjoiMC4wLjAwMDAiLCJQIjoiV2luMzIiLCJBTiI6Ik1haWwiLCJXVCI6Mn0%3D%7C0%7C%7C%7C&sdata=HnLM%2BPWHYkYX34vtXZdyL1gmGaEPrT5G3WYHW9cgqu0%3D&reserved=0)) and the following text:

“I request permission for the open-access journal PLOS ONE to publish XXX under the Creative Commons Attribution License (CCAL) CC BY 4.0 ([http://creativecommons.org/licenses/by/4.0/](https://eur01.safelinks.protection.outlook.com/?url=https%3A%2F%2Furl.za.m.mimecastprotect.com%2Fs%2FrJMLCY6YG5tLOQ8JqfjBvgJ&data=05%7C02%7Cmttdan008%40myuct.ac.za%7C323ec11e6bfa4539acb308dc49045d61%7C92454335564e4ccfb0b024445b8c03f7%7C0%7C0%7C638465530874773650%7CUnknown%7CTWFpbGZsb3d8eyJWIjoiMC4wLjAwMDAiLCJQIjoiV2luMzIiLCJBTiI6Ik1haWwiLCJXVCI6Mn0%3D%7C0%7C%7C%7C&sdata=csFWkA%2BRjYH8qOP0SkTBnYl%2FE%2FAp1QkPJxDb5st%2FZp0%3D&reserved=0)). Please be aware that this license allows unrestricted use and distribution, even commercially, by third parties. Please reply and provide explicit written permission to publish XXX under a CC BY license and complete the attached form.”

Please upload the completed Content Permission Form or other proof of granted permissions as an "Other" file with your submission.

In the figure caption of the copyrighted figure, please include the following text: “Reprinted from [ref] under a CC BY license, with permission from [name of publisher], original copyright [original copyright year].”

2. If you are unable to obtain permission from the original copyright holder to publish these figures under the CC BY 4.0 license or if the copyright holder’s requirements are incompatible with the CC BY 4.0 license, please either i) remove the figure or ii) supply a replacement figure that complies with the CC BY 4.0 license. Please check copyright information on all replacement figures and update the figure caption with source information. If applicable, please specify in the figure caption text when a figure is similar but not identical to the original image and is therefore for illustrative purposes only.

**Response: The figures were made using Biorender software under a paid institutional account. However, Biorender have declined to give us a CC BY 4.0 license authorisation; they could only offer CC-BY-NC-ND license.**

**Therefore, we have decided to replace the original figures with figures made using PowerPoint which is compatible with CC BY 4.0 copyright license. The arrangement of the figures and the information communicated thereof is our intellectual property therefore we have removed specific images that were solely available from Biorender and replaced them with generic images or images solely made by us. We hope this is sufficient.**

**Reviewers' comments:**
**Reviewer's Responses to Questions**

**Comments to the Author**
1. Does the manuscript provide a valid rationale for the proposed study, with clearly identified and justified research questions?
The research question outlined is expected to address a valid academic problem or topic and contribute to the base of knowledge in the field.

Reviewer #1: Yes

Reviewer #2: Yes

2. Is the protocol technically sound and planned in a manner that will lead to a meaningful outcome and allow testing the stated hypotheses?
The manuscript should describe the methods in sufficient detail to prevent undisclosed flexibility in the experimental procedure or analysis pipeline, including sufficient outcome-neutral conditions (e.g. necessary controls, absence of floor or ceiling effects) to test the proposed hypotheses and a statistical power analysis where applicable. As there may be aspects of the methodology and analysis which can only be refined once the work is undertaken, authors should outline potential assumptions and explicitly describe what aspects of the proposed analyses, if any, are exploratory.

Reviewer #1: No

Reviewer #2: Yes

**Response: Thank you for the feedback. In the revised manuscript we have included the specific descriptions of the cutoff points and the specific experiments to be conducted.**

3. Is the methodology feasible and described in sufficient detail to allow the work to be replicable?
Descriptions of methods and materials in the protocol should be reported in sufficient detail for another researcher to reproduce all experiments and analyses. The protocol should describe the appropriate controls, sample size calculations, and replication needed to ensure that the data are robust and reproducible.

Reviewer #1: No

Reviewer #2: Yes

**Response: Thank you for the feedback. In the revised manuscript we have included specifics of the experiments to be conducted and their replications where appropriate. We have also included the accepted power and sample size calculations.**

4. Have the authors described where all data underlying the findings will be made available when the study is complete?
The [PLOS Data policy](https://eur01.safelinks.protection.outlook.com/?url=https%3A%2F%2Furl.za.m.mimecastprotect.com%2Fs%2F4wsvCoYnqytrZPA4ZF2MV3E&data=05%7C02%7Cmttdan008%40myuct.ac.za%7Ce9646b67bf464ac0db3108dc43abda49%7C92454335564e4ccfb0b024445b8c03f7%7C0%7C0%7C638459652806631576%7CUnknown%7CTWFpbGZsb3d8eyJWIjoiMC4wLjAwMDAiLCJQIjoiV2luMzIiLCJBTiI6Ik1haWwiLCJXVCI6Mn0%3D%7C60000%7C%7C%7C&sdata=5hHlvBOhnAPYkt%2FTNIkcwJT7ZM8FEIoi9MpKYkKk%2BIg%3D&reserved=0) requires authors to make all data underlying the findings described in their manuscript fully available without restriction, with rare exception, at the time of publication. The data should be provided as part of the manuscript or its supporting information, or deposited to a public repository. For example, in addition to summary statistics, the data points behind means, medians and variance measures should be available. If there are restrictions on publicly sharing data—e.g. participant privacy or use of data from a third party—those must be specified.

Reviewer #1: No

Reviewer #2: Yes

**Response: We have included a data availability statement. Lines 519 – 521 of the revised manuscript.**

5. Is the manuscript presented in an intelligible fashion and written in standard English?
PLOS ONE does not copyedit accepted manuscripts, so the language in submitted articles must be clear, correct, and unambiguous. Any typographical or grammatical errors should be corrected at revision, so please note any specific errors here.

Reviewer #1: Yes

Reviewer #2: Yes

**Response: The revised clean manuscript has been reviewed, proofread, and the language edited.**

6. Review Comments to the Author
Please use the space provided to explain your answers to the questions above and, if applicable, provide comments about issues authors must address before this protocol can be accepted for publication. You may also include additional comments for the author, including concerns about research or publication ethics.
You may also provide optional suggestions and comments to authors that they might find helpful in planning their study.
(Please upload your review as an attachment if it exceeds 20,000 characters)

Reviewer #1: In the manuscript titled "A Study Protocol to Characterize Pathophysiological and Molecular Markers of Rheumatic Heart Disease and Degenerative Aortic Stenosis Using Multiparametric Cardiovascular Imaging and Multi-omics Techniques," the authors outline a protocol for patient recruitment and the rationale for conducting the study. This initiative is commendable, especially in light of the latest recommendations for open science, which emphasize the importance of publishing study protocols before conducting research and subsequently sharing the results. However, several significant modifications are necessary before publication and, crucially, before initiating the study.

**Response: Thank you for taking the time to review our manuscript and for your kind and constructive feedback.**
1. **Inclusion Criteria:** The specified age range of 18-80 years is overly broad, considering the significant physiological differences across younger, middle-aged, and older patients. This criterion should be reassessed, or at the very least, patients should be categorized into distinct age groups as recommended, ensuring adequate representation in each category.

**Responses: Thank you for the feedback. After careful consideration, we have revised the upper age limit to 70 years. The mean age of the patients currently recruited in the study is 44.7±13.7 years for RHD patients and 64.2±12.8 years for degenerative AS patients. We acknowledge that the range is larger than desirable, especially when age is a frequent confounder in such studies. However, degenerative AS is often related to advanced age. Furthermore, at the location where we are recruiting the participants, most of the patients present for valve replacement at the advanced stages of the disease (both RHD and AS) which also tends to make them older than in other populations.**

**Given the larger sample sizes we are now looking to recruit, we believe that there should be sufficient age stratification to enable a confounding factor of age to be appropriately accounted for.**
2. **Sex Differences:** The protocol currently overlooks the critical aspect of sex differences in cardiovascular disease (CVD) pathology. Given the historical underrepresentation of females in CVD research, it is imperative to aim for gender-balanced groups to address this gap effectively.

**Response: Thank you for highlighting this information. We have added a statement indicating that we shall ensure that there is a balance between males and females in the studied groups. Lines 260-261.**
3. **Sample Size:** The document does not specify the number of participants for each group, a critical omission. While calculating the power of multi-omics studies may be challenging, it is still necessary to provide power calculations or at least an estimate of the minimum number of subjects required for each omics approach to ensure reliable results.

**Response: Thank you for your suggestions. After reconsideration and analysis, we have increased the sample size to 250. This is based on the information obtained from using available tools for power calculation for metabolomics and genomics studies. In the revised manuscript we have included the justification for using a sample size of 250 VHD participants and an equal number of controls. We have also broken down the sample size per disease group and categories.**
4. **Sample Collection and Processing:** Detailed information on blood and tissue sample collection and processing is lacking. This should include the types and brands of collection tubes, the number of tubes for serum/plasma, tube volume, anticoagulants used, centrifugation speed and conditions for serum/plasma separation, time from collection to storage, aliquot numbers, and storage conditions. For transcriptomics, considering the variability in platelet counts among patients, which can significantly affect results, platelet-poor plasma may be preferable for miRNA analysis. Details on RNA preservation and extraction methods for both serum/plasma and tissue samples, including the use of RNA later, Trizol, or snap freezing with liquid nitrogen, should be provided.

**Response: In the revised manuscript we have added the detailed information for sample collection and processing. We have also added some detailed information on miRNA extraction and handling.**
5. **Open Research Data Initiative and FAIR Principles:** The manuscript should also address the Open Research Data Initiative and incorporate the FAIR (Findable, Accessible, Interoperable, and Reusable) principles. Discussing how the study's data will be made available in accordance with these principles is essential. This includes detailing the data management plan, which should outline how data will be stored, shared, and made accessible to ensure that it is discoverable and usable by others in the research community. Highlighting the commitment to these practices not only aligns with the push towards open science but also enhances the transparency, reproducibility, and impact of the research.

**Response: We have added a data availability statement in the revised manuscript. Lines 548-550.**
The considerations and suggestions from the referenced paper ([https://doi.org/10.1016/j.molmed.2023.09.004](https://eur01.safelinks.protection.outlook.com/?url=https%3A%2F%2Furl.za.m.mimecastprotect.com%2Fs%2FoYaiCpgo02fnKOgDKsvU_W1&data=05%7C02%7Cmttdan008%40myuct.ac.za%7Ce9646b67bf464ac0db3108dc43abda49%7C92454335564e4ccfb0b024445b8c03f7%7C0%7C0%7C638459652806636767%7CUnknown%7CTWFpbGZsb3d8eyJWIjoiMC4wLjAwMDAiLCJQIjoiV2luMzIiLCJBTiI6Ik1haWwiLCJXVCI6Mn0%3D%7C60000%7C%7C%7C&sdata=n8vwpWOTpwMLlcbVFS1EVu8d4bxwhjnO4coVyC81s20%3D&reserved=0)) provide valuable guidance on designing and conducting multi-omic studies. While direct citation is not needed, employing this resource as a framework for study design and execution can greatly enhance the protocol's robustness and scientific contribution.

**Response: Thank you for the suggestion. The article was very informative.**

Reviewer #2: The current study represent an interesting topic to study and to conduct.

**Response: Thank you for taking your time to review our manuscript and for your kind and positive feedback.**

We have few comments:
1- There is a need to further support the current proposed sample size (120 pts).

**Response: Thank you for the feedback. We have increased the sample size to 250 participants, and we have also included a justification for the sample size in the revised manuscript.**

2- Clarify properly if you recruit the patients from endemic area of RHD or not. The current status of RHD.

**Response: We are recruiting in RHD endemic area, the current prevalence of RHD is 29.40/100,000 as at 2017.**

3- Justify how your cohort would represent a real-world cohort regarding RHD and AS.

**Response: We are a little confused by your answer/request. This population represents a real-world cohort in that it is reflective of the patients that we are regularly treating daily in Cape Town hospitals. In this respect, being able to distinguish between AS and RHD where the prevalence of both is high becomes an important clinical question.**

4- High light your measures to avoid bias, selection bias.

**Response: Since this is a case-control study we have defined clear inclusion criteria that will be used to recruit patients into RHD or AS group and the healthy controls.**

5- Discuss the expected major limitations or challenges.

**Response: Thank you for the suggestion. In the revised manuscript we have highlighted some potential limitations and challenges to the study. Lines 524 – 531.**

Regards

7. PLOS authors have the option to publish the peer review history of their article ([what does this mean?](https://eur01.safelinks.protection.outlook.com/?url=https%3A%2F%2Furl.za.m.mimecastprotect.com%2Fs%2FA-auCqjpvZS8lkgWlcYBVYp&data=05%7C02%7Cmttdan008%40myuct.ac.za%7Ce9646b67bf464ac0db3108dc43abda49%7C92454335564e4ccfb0b024445b8c03f7%7C0%7C0%7C638459652806641820%7CUnknown%7CTWFpbGZsb3d8eyJWIjoiMC4wLjAwMDAiLCJQIjoiV2luMzIiLCJBTiI6Ik1haWwiLCJXVCI6Mn0%3D%7C60000%7C%7C%7C&sdata=VTVyvhWj8goXabc4vXdc4kVvAAR7B3rbVts41TO69XA%3D&reserved=0)). If published, this will include your full peer review and any attached files.
If you choose “no”, your identity will remain anonymous but your review may still be made public.
**Do you want your identity to be public for this peer review?** For information about this choice, including consent withdrawal, please see our [Privacy Policy](https://eur01.safelinks.protection.outlook.com/?url=https%3A%2F%2Furl.za.m.mimecastprotect.com%2Fs%2FVnQwCr0qw9i8ony9oc64BQW&data=05%7C02%7Cmttdan008%40myuct.ac.za%7Ce9646b67bf464ac0db3108dc43abda49%7C92454335564e4ccfb0b024445b8c03f7%7C0%7C0%7C638459652806647298%7CUnknown%7CTWFpbGZsb3d8eyJWIjoiMC4wLjAwMDAiLCJQIjoiV2luMzIiLCJBTiI6Ik1haWwiLCJXVCI6Mn0%3D%7C60000%7C%7C%7C&sdata=3zwcCGsDP47PZ3YushUPY3iBNkAMe0ZlPw%2FMVc2LLFk%3D&reserved=0).

Reviewer #1: No

Reviewer #2: **Yes:** Rami Riziq Yousef Abumuaileq

I look forward to your positive consideration and hearing from you.

Yours sincerely,

Ntobeko Ntusi

Professor of Medicine and Head of Department
